# The Influence of Modernization and Disease on the Gastric Microbiome of Orang Asli, Myanmars and Modern Malaysians

**DOI:** 10.3390/microorganisms7060174

**Published:** 2019-06-14

**Authors:** Eng Guan Chua, Mun Fai Loke, Selva Perumal Gunaletchumy, Han Ming Gan, Kavitha Thevakumar, Chin Yen Tay, Sylvia Young, Than Than Aye, Win Win Maw, Mya Mya Aye, Alex Hwong-Ruey Leow, Ahmad Najib Azmi, Sri Ganesh Kalimuthu, Haji Mohd Akmal Bin Dato Dahlan, Khean Lee Goh, Jamuna Vadivelu

**Affiliations:** 1 The Marshall Centre for Infectious Diseases Research and Training, University of Western Australia, Perth, WA 6009, Australia; alfred.tay@uwa.edu.au; 2 Department of Medical Microbiology, University of Malaya, Kuala Lumpur 50603, Malaysia; lmunfai@gmail.com (M.F.L.); gl_2512@hotmail.com (S.P.G.); kavitha01um@gmail.com (K.T.); jamuna@ummc.edu.my (J.V.); 3 School of Life Sciences and Chemical Technology, Ngee Ann Polytechnic, Singapore 599489, Singapore; 4 School of Science, Monash University Malaysia, Selangor 47500, Malaysia; han.gan@deakin.edu.au; 5 Centre for Integrative Ecology, School of Life and Environmental Sciences, Deakin University, Melbourne, VIC 3216, Australia; 6 Harry Perkins Institute of Medical Research, The Centre for Medical Research at the University of Western Australia, Perth, WA 6009, Australia; sylvia.young@uwa.edu.au; 7 Department of Gastroenterology, Thingangyun Sanpya General Hospital, Yangon 11071, Myanmar; ttaye06@gmail.com; 8 Microbiology Department, University of Medicine 2, Yangon 11031, Myanmar; winwinmawizumo@gmail.com; 9 University of Medical Technology, Mandalay 05071, Myanmar; prof.myamyaaye.umtm@gmail.com; 10 Department of Medicine, University of Malaya, Kuala Lumpur 50603, Malaysia; hrleow@ummc.edu.my (A.H.-R.L.); drahmadnajib@gmail.com (A.N.A.); klgoh56@gmail.com (K.L.G.); 11 CliniPath Malaysia Sdn Bhd, Kuala Lumpur 55100, Malaysia; fahmie@mgrc.com.my; 12 Hospital Orang Asli Gombak, Selangor 53100, Malaysia; drakmal.dahaman@moh.gov.my

**Keywords:** *Helicobacter pylori*, *16S rRNA* gene sequencing, Orang Asli, Myanmar, Malaysian, gastric disease, beta diversity, microbiome

## Abstract

The present study explored the differences in gastric microbiome between three distinct populations of Southeast Asia. These include the isolated Orang Asli population and modern Malaysians, as well as patients from Myanmar, the least developed country in the region. All 79 subjects recruited in this study had *Helicobacter pylori* infection. Based on alpha diversity analysis, Orang Asli had the richest and most diverse gastric microbiome, followed by Myanmar and modern Malaysian groups. Beta diversity analysis revealed significant separation of samples between different populations. These observations are likely to be associated with the level of modernization of each population. Our data further suggested increased bacterial species richness and diversity of the gastric microbiome in individuals who were less modernized, particularly in the Orang Asli group, could suppress the growth of *H. pylori*. In addition, there were significant variations in the gastric microbiome between modern Malaysians with different types of gastric diseases. Notably, *Cutibacterium acnes* was present at significantly greater abundance level in patients with non-ulcerative dyspepsia than those with peptic-ulcer diagnosis. This suggests that *C. acnes* may also play a role in gastritis besides *H. pylori*, which merits further investigation.

## 1. Introduction

The human body does not contain only our own cells but is also a host to numerous microorganisms. To illustrate this scenario, Joshua Lederberg coined the term “microbiome” to signify “the ecological community of commensal, symbiotic, and pathogenic microorganisms that literally share our body space” [1]. Up to 60% of human-associated microbes could not be cultured in vitro [2,3,4], leading to substantial underestimation of the actual microbiota diversity in different parts of human body. For years, the study of the gastric microbial composition was stagnated as the human stomach was considered a sterile organ due to its hostile acidic environment [5]. However, the discovery of *Helicobacter pylori* in 1982 relinquished this central dogma [6].

A recent study conducted on two populations in Colombia found striking differences in microbiome composition between the two populations, which could be related to dietary differences and gastric cancer risk [7]. This supports the need for population-based microbiome studies to better understand how changes related to lifestyle, diet, socioeconomic level and disease status could alter the microbiota, and if possible, to revert or mimic such changes for better human health. To date, while there have been studies comparing the compositions of gut microbiota in populations of different ethnic and geographic origins [8,9,10,11], such studies are generally lacking in Southeast Asia.

Malaysia and Myanmar are both multiracial and multicultural countries located in Southeast Asia. In Malaysia, the major populations are Malay, Chinese and Indian, while Myanmar’s primary residents consist of Burman, Shan, Karen, Rakhine, Chinese, Indian and Mon. Historically, Myanmar and Malaysia were located along the Maritime Silk Road and were once colonized by the British until 1948 and 1957, respectively. Since then, while Malaysia continues to advance, Myanmar civilization, under the isolationistic policies of its military government from 1962 to 2011, had remained stagnant [12]. The level of modernization in these two major populations can be reflected by their access to clean drinking water and sanitary facilities, which are both vital development indicators. Until 2015, only 68% and 64.7% of households in the less-developed Myanmar nation had access to clean drinking water and basic sanitation service [13]. In contrast, 96% and 99.6% of Malaysian households were provided with clean water supply and adequate sanitation, respectively.

Orang Asli are the indigenous minorities residing in Peninsular Malaysia. More importantly, they are the oldest inhabitants of Peninsular Malaysia. The Orang Asli population is a collective of 18 ethnic subgroups further classified under Negrito, Senoi and Proto-Malay for administrative purposes [14]. While the Negrito comprises the Kensiu, Kintaq, Jahai, Lanoh, Mendriq and Bateq subethnic groups, classified under the Senoi are the Semai, Temiar, Jah Hut, Semaq Beri, Che Wong and Mah Meri subgroups. Proto-Malay, on the other hand, contains the Temuan, Temoq, Semelai, Jakun, Orang Kuala, Orang Kanaq and Orang Seletar subpopulations [15]. Orang Asli mostly live in remote mountainous areas, away from urbanization with no access to clean water supply and proper sanitary service [16]. Each subgroup can be found within a specific geographical space usually next to a river valley and isolated from the others.

This study highlighted the differences in gastric microbiome of three distinct populations from the Southeast Asia region (the isolated Orang Asli of Malaysia, Myanmar residents and modern Malaysians), which is likely associated with the level of modernization. We further demonstrated the influence of gastric diseases on human gastric microbiome, albeit interestingly, only in modern Malaysian patients.

## 2. Materials and Methods

### 2.1. Ethics Statement

This study was approved by the National Institutes of Health of Malaysia Clinical Research Centre and Medical Research & Ethics Committee (NMRR-12-671-11909), University of Malaya Medical Centre (UMMC) Medical Ethics Committee (MEC Ref. No. 1023.3) and the Ethics and Research Committee of the University of Medicine 2, Myanmar. Gastric biopsy samples were obtained with informed and written consent from subjects who presented for endoscopy at UMMC and Thingangyun Sanpya General Hospital.

### 2.2. Screening of H. pylori Infections in Orang Asli

Between 28 October and 12 December 2013, 177 asymptomatic volunteers from five different locations: (1) Kampung Sungai Padi, Lipis district, Pahang state; (2) Kampung Donglai, Hulu Langat district, Selangor state; (3) Cameron Highlands district, Pahang state; (4) Gua Musang district, Kelantan state and; (5) Gombak district and Selangor state, were tested for *H. pylori* infection. Among these 177 volunteers; 69, 45, 4 and 1 were from the Temiar, Semai, Mah Meri and Jah Hut subethnic groups classified under the Senoi group, respectively; 56 and 1 were from the Temuan and Jakun subgroups derived from the Proto-Malay group, respectively; and a remaining volunteer who was of Iban origin that does not belong to any of the three primary Orang Asli tribal groups. The test was conducted using the PYtest 14C-urea breath test (UBT) kit (Tri-Med, West Leederville, WA, Australia). Breath samples were analyzed using the microCOUNT Lite Liquid Scintillation Counter. Results were reported as disintegrations per minute (DPM). A DPM count of less than 50 was interpreted as a negative result while DPM counts in the range of 50–199 were classified as indeterminate. A DPM count of 200 and above was considered positive for *H. pylori* infection.

### 2.3. Collection of Gastric Biopsies

Of the 79 gastric tissue biopsy samples collected for this study, from September 2011 to June 2014, 10 and 32 were from UBT-positive; Orang Asli volunteers and non-Orang Asli Malaysian patients who were symptomatic and previously tested positive for *H. pylori* infection, respectively. These individuals underwent endoscopic examination followed by the collection of gastric biopsies at UMMC’s Endoscopy Unit. The remaining 37 samples were collected from Myanmar patients at the Department of Gastroenterology of Thingangyun Sanpya General Hospital, Yangon, Myanmar. All subjects were further diagnosed with gastric cancer (GC), peptic ulcer disease (PUD) or non-ulcerative dyspepsia (NUD) based on endoscopy evaluation.

Biopsy specimens were obtained from the gastric antrum and fundus for metagenomics purposes, CLOtest rapid urease testing, *H. pylori* culturing, and histopathological examination. Culturing of *H. pylori* isolate was not performed on Myanmar patient samples in this study. Histopathological examination was performed independently by Dr. Sri Ganesh Kalimuthu of the CliniPath Malaysia Sdn Bhd.

### 2.4. Illumina 16S rRNA Gene Sequencing

Genomic DNA was extracted from gastric biopsy samples using MasterPure^TM^ DNA purification kit (Epicentre, Madison, WI, USA) according to manufacturer’s instructions. The V3–V4 region of *16S rRNA* gene was amplified using S-D-Bact-0341-b-S-17 and S-D-Bact-0785-a-A-21 primers designed to include the Illumina-compatible adaptors [17,18]. The 16S amplicon libraries were prepared according to Illumina 16S library preparation protocol [19]. Initial PCR amplification was performed using NEBNext High-Fidelity Master Mix (New England Biolabs, Ipswich, MA, USA) with the following conditions: An initial denaturation at 98 °C for 30 s, followed by 30 cycles consisting of denaturation (98 °C for 10 s), annealing (60 °C for 2 min) and extension (72 °C for 20 s), and a final extension step at 72 °C for 1 min. Automated cluster generation and a 2 × 250 bp paired-end sequencing (MiSeq 500-cycle reagent kit V2) was carried out on the MiSeq platform (Illumina, San Diego, CA, USA) at the Monash University Malaysia Genomics Facility. Metagenomics data reported in this paper are available for public access through the MG-RAST server. The accession number and demographic characteristics of each sample are listed in Table S1.

### 2.5. Data Analysis

The 16S sequencing data were quality-trimmed using Sickle (https://github.com/najoshi/sickle) using the following parameters: –q 20 –l 200. Merging of overlapping paired-end sequences was performed using MeFit with default parameters [20]. Filtering of chimeric sequences, *de novo* greedy clustering of *16S rRNA* sequences into Operational Taxonomic Units (OTUs) at 97% similarity threshold, and removal of singleton and chimeric OTUs were conducted using Micca (version 1.7.0) [21]. Taxonomic assignment of the representative OTUs was performed using the Bayesian LCA-based taxonomic classification method with a 1^−100^ cut-off e-value and 100 bootstrap replications, against NCBI 16S microbial database [22]. Taxonomic assignment at each level was accepted with a minimum confidence score of 80. The OTU table including taxonomic information is available in Table S2. Unclassified OTUs were retained and included in all following analyses.

Microbial diversity analysis was performed on rarefied OTU abundance matrix (depth value of 17358) using QIIME (version 1.9.1) [23]. Alpha diversity was evaluated based on the following metrics: Observed species and Shannon diversity indices. A non-parametric two-sample *t*-test with Bonferroni correction was used to compare the alpha diversity metrics between different groups. Principle coordinate analysis (PcoA) using Bray–Curtis and Jaccard distance metrics was performed to visualize separation of samples. Non-parametric statistical analysis of the distance metric was performed using ANOSIM with 1000 permutations. Plots were generated using PhyloToAST software [24]. Prediction of KEGG functional pathways was conducted using Piphillin and the results are available in Table S3 [25,26]. Pearson correlations between KEGG pathways and bacterial genera were examined using microbiomeSeq R package (https://github.com/umerijaz/microbiomeSeq.git). Only negative and positive correlations of ≤−0.8 and ≥0.8, respectively, and with *p*-values less than 0.01, were considered.

### 2.6. Statistical Analysis

Bacterial genera and KEGG functional pathways with minimum average relative abundances of 0.1% were compared between groups by Wilcoxon rank-sum testing, followed by Bonferroni correction. Differences with adjusted *p*-values of less than or equal to 0.01 were considered significant.

## 3. Results

### 3.1. H. pylori Prevalence among Orang Asli

Among 177 Orang Asli subjects who were screened for *H. pylori* infection, only 18 (10.2%) and 53 (30.3%) were tested positive and negative, respectively. The rest, interestingly, had indeterminate UBT readings. The demographics and UBT results of the Orang Asli subjects in this study are summarized in Table 1. Ten UBT-positive Orang Asli individuals consented for further endoscopy examination and provided gastric biopsies for this study.

### 3.2. The Gastric Microbial Community of H. pylori-Positive Orang Asli, Myanmar and Modern Malaysian Subjects

The general characteristics and clinical diagnosis of study subjects who underwent endoscopic examination are summarized in Table 2. A total of 22,526,214 reads were generated for all 79 samples. Following quality trimming and merging of overlapping paired-end sequences, 9,912,950 sequences with an average length of 454.3 ± 8.1 bp were retained. The number of sequences per sample ranged from 20,042 to 233,069 (median 115,971). Of 2233 OTUs acquired via *de novo* clustering algorithm, 1466 and 1105 were taxonomically assigned down to and phylum and genus level, respectively, with an 80% confidence threshold, which were subsequently aggregated to 20 bacterial phyla and 443 bacterial genera, respectively, for further analysis.

**Figure 1:**
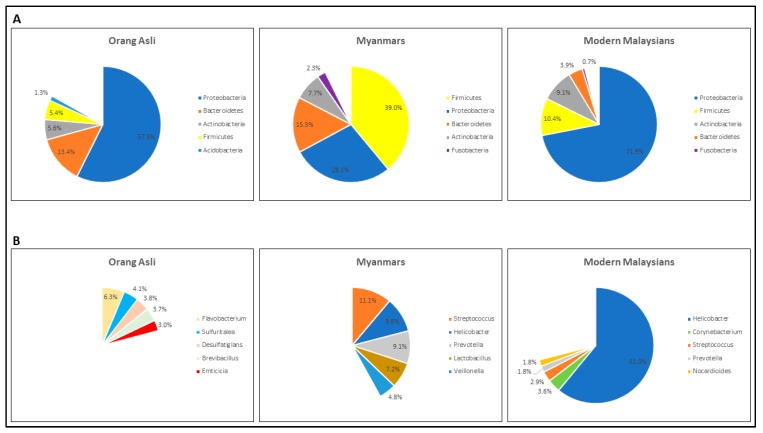
Bacterial phyla and genera compositions in Orang Asli, Myanmar and modern Malaysian non-ulcerative dyspepsia (NUD) patients. (**A**) Mean relative abundances of the five most predominant bacterial phyla in each population. (**B**) Mean relative abundances of the five most predominant bacterial genera in each cohort.

As our study subjects differed not only by population but also by disease status, a comparison of those with only NUD was therefore performed to exclude the influence of PUD and GC when examining the microbial differences between three major populations including Orang Asli, modern Malaysians comprising Chinese, Indian and Malay ethnic groups, and Myanmar subjects. Overall, Proteobacteria, Firmicute and Actinobacteria constituted the three most predominant phyla, at 71.9%, 10.4% and 9.1% of relative abundances (Figure 1A), respectively, in the gastric microbiome of modern Malaysian patients. Similarly, in the Orang Asli samples, while having both Proteobacteria and Actinobacteria as the first and third most abundant phyla, at 57.3% and 5.6%, respectively, Bacteroidetes, not Firmicute, was the second most common bacterial phylum (13.4%) identified. In Myanmar patients, the top three most abundant phyla were Firmicutes, Proteobacteria and Bacteroidetes, at 39%, 28.1% and 15.5%, respectively.

At the genus level, *Helicobacter* was the most abundant bacterial genus identified in the modern Malaysians, with a relative abundance of 61% (Figure 1B). In both Myanmar and Orang Asli patients, however, the *Helicobacter* bacterial loads were only 9.8% and 0.25%, respectively. While a high abundance of *Lactobacillus* (7.2%) was detected in Myanmar patients, Orang Asli group contained mostly environmental bacterial genera including *Flavobacterium*, *Sulfuritalea*, *Desulfatiglans*, *Brevibacillus* and *Massilia*.

**Figure 2:**
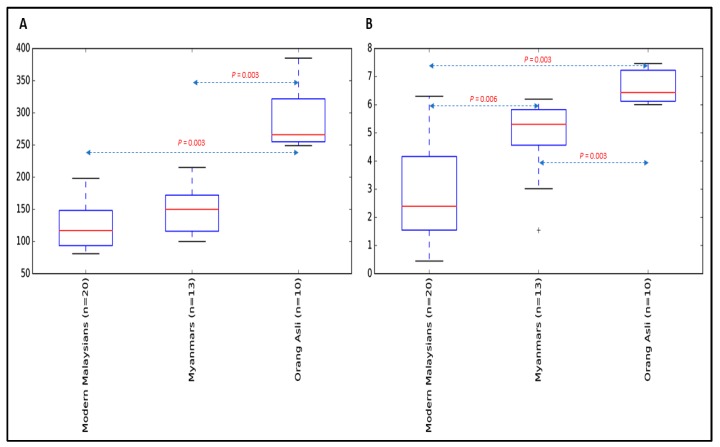
Alpha diversity analysis between Orang Asli, Myanmar and modern Malaysian groups diagnosed with NUD. (**A**) Alpha diversity estimation based on species richness index. (**B**) Alpha diversity estimation based on Shannon diversity index.

**Figure 3:**
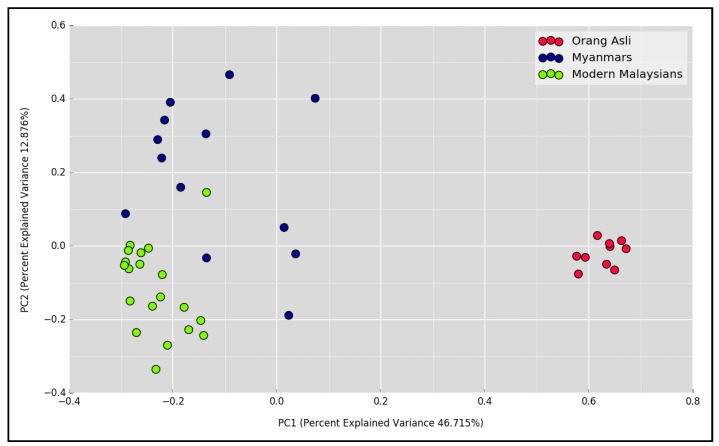
Beta diversity analysis between Orang Asli, Myanmar and modern Malaysian groups diagnosed with NUD. Principal coordinate analysis (PCoA) plot based on Jaccard index (*R* = 0.75, *p* < 0.001).

We next analyzed the diversity of bacterial populations amongst Orang Asli, Myanmar and modern Malaysian samples. Alpha diversity based on species richness and Shannon diversity indices was estimated. Both modern Malaysian and Myanmar groups had significantly reduced microbial richness (Figure 2A) and diversity (Figure 2B) relative to Orang Asli. It is worth mentioning that the gastric microbial community of modern Malaysians was the least diverse among all. To assess bacterial community differences between groups, principal coordinate analysis (PCoA) based on Jaccard metric was performed. The analysis revealed significant dissimilarities in microbial composition among the groups (Figure 3, *R* = 0.75, *p* < 0.001).

To identify distinguishing bacterial genera between Orang Asli, Myanmar and modern Malaysian NUD samples, 76 genera with minimum average abundances of 0.1% were subjected to Wilcoxon rank-sum testing followed by Bonferroni correction. Of 28 significant genera identified, as summarized in Table 3, 17 were profoundly enriched in the Orang Asli gastric microbiome compared to that of Myanmar and modern Malaysian samples. While *Nocardioides* was nearly absent in the Myanmar samples, modern Malaysians had the highest *Helicobacter* bacterial load, significantly greater than that of Orang Asli and Myanmar cohorts.

Functional prediction using Piphillin revealed 306 KEGG pathways (Table S3). Following rarefaction and filtering non-NUD samples, we then compared between different populations the pathways with total average abundances of at least 0.1% and considered only those with a minimum relative fold change of 1.25 and a Bonferroni-adjusted p-value of less than or equal to 0.01 as statistically significant. Seventy-six pathways were significant (Table S4). Notably more than one third of pathways (28/76) were significantly up- or down-regulated in Orang Asli samples in comparison to both Myanmar and modern Malaysian cohorts. Of 28 significantly differential pathways identified in Orang Asli, half were involved in metabolism of amino acids and organic compounds like ascorbate and aldarate, histidine, tryptophan, inositol phosphate, arginine and proline, phenylalanine, tyrosine and beta-alanine, which had positive significant association with *Bosea*, *Brevundimonas*, *Emticicia*, *Sphingorhabdus*, *Tardiphaga* and *Variovorax*; six environmental bacterial genera that were found present almost exclusively in the Orang Asli population (Table S5). Compared to both Orang Asli and Myanmar cohorts, significant up-regulation of FoxO signaling was observed in the modern Malaysian samples and was shown to be positively and negatively associated with *Helicobacter* and *Streptococcus*, respectively.

### 3.3. Gastric Diseases Altered the Microbial Community Structure in Modern Malaysian Patients

**Figure 4:**
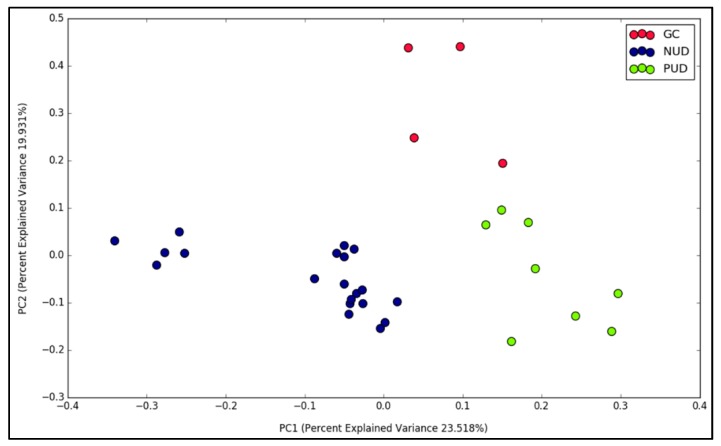
Beta diversity analysis between modern Malaysians with gastric cancer, peptic ulcer disease and non-ulcerative dyspepsia. PCoA plot based on Jaccard index (*R* = 0.4, *p* = 0.002).

We attempted additional diversity analysis to determine whether different gastric disease outcomes would affect the structural composition of gastric microbial community. The analysis was performed separately for each population to eliminate the confounding effect of population factor on disease status. Orang Asli group was excluded as NUD was the only gastric disorder diagnosed in this population. While clear separations between NUD, PUD and GC samples were observed in modern Malaysian samples (Figure 4, Jaccard index, *R* = 0.4, *p* = 0.002), interestingly, no evident grouping of Myanmar samples according to disease types could be observed.

**Figure 5:**
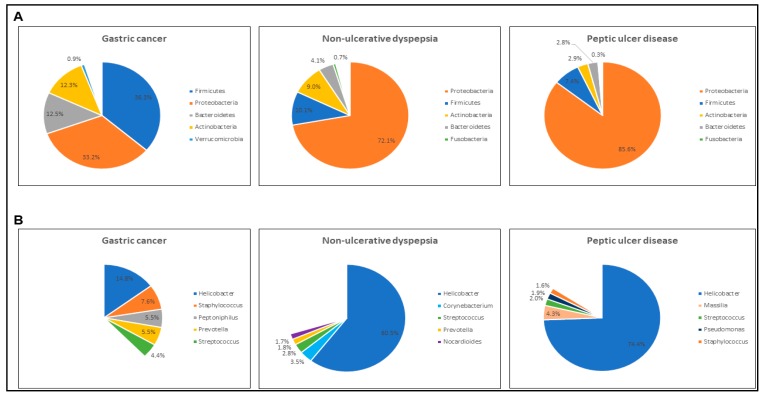
Bacterial phyla and genera compositions in modern Malaysians with gastric cancer, peptic ulcer disease and non-ulcerative dyspepsia. (**A**) Mean relative abundances of the five most dominating bacterial phyla in each disease group. (**B**) Mean relative abundances of the five most dominating bacterial genera in each disease group.

Both NUD and PUD samples shared similar major bacterial phyla compositions with Proteobacteria and Firmicutes being the two most predominant phyla, at 72.1% and 10.1% in the former and 85.6% and 7.4% in the latter (Figure 5). The GC samples, however, had comparable abundances of both Proteobacteria and Firmicutes, at 33.2% and 36.3%, respectively. At genus level, while *Helicobacter* bacteria constituted 60.5% and 74.4% of gastric microbiome in both NUD and PUD groups, respectively, in GC group the total abundance of top five most predominant genera including *Helicobacter* was 37.8%, indicating that GC samples had a more diverse and complex gastric bacterial population.

Due to small GC sample size (*n* = 4), comparison of bacterial genera abundances by Wilcoxon ranked-sum testing was performed only between NUD and PUD groups. Following Bonferroni correction, of 55 genera tested, only one appeared significant, which is *Cutibacterium* (adjusted *p* = 0.005, median abundances of 0.159% and 0.002% for NUD and PUD, respectively).

## 4. Discussion

In this study, the gastric microbial compositions of three distinct populations, Orang Asli, Myanmars and modern Malaysians, who all had *H. pylori* infection based on positive UBT result, were investigated. It is important to note that the median abundance of *Helicobacter* bacteria in Orang Asli was merely 0.25%, which is several orders of magnitude less than that of modern Malaysian and Myanmar individuals. This is likely due to the significantly more diverse bacterial makeup in the gastric environment of Orang Asli compared to that of modern Malaysians and Myanmars. Although the human stomach serves as the primary reservoir for *H. pylori* colonization, the findings above indicate that the degree of colonization can be affected by the changes in the gastric microenvironment. It is evident that the presence of environmental bacterial species in the stomach of Orang Asli, as well as in the Myanmar individuals to a certain extent but less, could suppress the growth of *H. pylori*, while the lack of these foreign environmental isolates in modern Malaysians allows *H. pylori* to flourish. This finding could possibly explain why most Orang Asli individuals had a borderline UBT outcome, presuming most of them are likely to be *H. pylori*-positive.

In both Orang Asli and modern Malaysian groups, Proteobacteria was found to be the predominant bacterial phylum. While Proteobacteria in modern Malaysians contained primarily *Helicobacter* bacteria belonging to the class Epsilon Proteobacteria, Orang Asli harbored mostly environmental microbes of Alpha-, Beta-, Delta- and Gamma-proteobacteria classes. Notably, the Orang Asli gastric microbiome had a Firmicutes/Bacteroidetes ratio of 0.41, which is at least 6-fold less than that of Myanmar and modern Malaysian groups, at 2.51 and 2.65, respectively. It was previously shown that a high-fat/high-sugar diet promotes the growth of Firmicutes while reducing the abundance of Bacteroidetes in the distal gut of rodents [27]. Additionally, when an obese individual shifted to a fat-restricted diet or a carbohydrate-restricted low-calorie diet, the abundance levels of Bacteroidetes and Firmicutes were increased and decreased, respectively [28]. Together, these observations suggest that both modern Malaysian and Myanmar populations are likely to consume a diet high in carbohydrate and/or fat content possibly attributed to influx of Western culture as part of modernization process, while Orang Asli are still maintaining traditional plant-based diets low in fat and carbohydrate corresponding to their hunting/planting lifestyle [29].

Of the several bacterial genera that were significantly enriched in Orang Asli, *Bosea*, *Sphingorhabdus*, and *Variovorax,* they also displayed significant positive correlations to arginine and proline metabolism, and β-alanine metabolism, respectively. While a diet supplemented with arginine and proline was shown to improve the wound healing process in diabetic rats [30], β-alanine supplementation as reported in a recent meta-analysis was able to improve exercise performance [31]. It is thought that these bacteria may facilitate the metabolism and uptake of these amino acids that are widely available in vegetables and grains consumed by Orang Asli, which is then likely to increase the wound healing and physical capacities in Orang Asli to enhance their survival fitness in an outback setting.

While bacteria of the genus *Nocardioides* was found in nearly all modern Malaysian (95%, 19/20) and Orang Asli (100%, 10/10) samples, it was detected in only 23.1% (3/13) of Myanmar samples. Members of the genus *Nocardioides* are mostly environmental bacteria found in soil or plants and only one *Nocardioides* species has thus far been isolated from a human faecal sample [32]. The high prevalence of *Nocardioides* species could be a distinct feature of gastric microbiome in peninsular Malaysians including Orang Asli, albeit the influence of this *Nocardioides* species on human health is uncertain.

Contrary to modern Malaysian samples, the Myanmar population did not show distinct clustering of samples by gastric disease conditions. It is thought that lifestyle and dietary habits, geographical and socioeconomic factors, and/or host genetics may have greater influence on the gastric microbiome than disease conditions in the Myanmar population. In modern Malaysians, PUD samples harbored the most *H. pylori*, which is expected as this bacterium is an important driving factor for peptic ulcer disease outcome [33]. Notably, NUD samples had significantly more *Cutibacterium* bacteria than in the PUD samples. At the species level, it was further identified as *C. acnes*, formerly known as *Propionibacterium acnes* [34]. Interestingly, while *H. pylori* has been known for its dominant role in causing different gastric disorders, *C. acnes* was identified as a possible causative agent for lymphocytic gastritis [35]. It is important to clarify that NUD, also known as functional dyspepsia, is the preferred clinical diagnosis term assigned to patients with gastritis, on the basis of endoscopic and histological results [36]. To examine the potential role of *C. acnes* in the development of gastric diseases alongside *H. pylori*, further investigation is required. This would help to expand our current knowledge of bacterium-driven gastric diseases.

## 5. Conclusions

This study highlighted the taxonomic differences between the gastric microbiome of three distinct populations from Southeast Asia including Orang Asli, Myanmars and modern Malaysia, and such differences are possibly due to different levels of modernization. Distinguishing gastric microbial community structure could also be seen between different gastric disease conditions in modern Malaysians, albeit not in Myanmar patients. Importantly, the gastric microbiome of Orang Asli, who lived in isolation from urbanization, and a near primitive lifestyle with no use of any modern antibiotics and few modern medicines, may represent one of the most “original” snapshots of human gastric microbiome to-date.

## Figures and Tables

**Table 1:** Urea breath test results and demographics of Orang Asli subjects.

Origin	*N*	Positive	Indeterminate	Negative	Mean Age (years)	Married (%)	Female (%)
Temiar	69	3	49	17	33 (16–80)	85%	67%
Temuan	56	3	32	21	29 (15–56)	69%	51%
Semai	45	11	19	15	32 (14–60)	83%	77%
Others (Mah Meri, Iban, Jah Hut and Jakun)	7	1	6	0	39 (27–53)	43%	57%

**Table 2:** Demographics and clinical findings of study subjects who underwent endoscopic examination.

Country	Ethnic Group	N	Mean Age (years)	NUD	PUD	GC
Malaysia	Orang Asli (Semai)	3	33.0	3	0	0
	Orang Asli (Temiar)	7	32.3	7	0	0
	Chinese	16	47.9	7	6	3
	Indian	10	34.6	8	1	1
	Malay	6	37.5	5	1	0
	**Overall**	**42**	**39.6**	**30**	**8**	**4**
Myanmar	Burmese	22	48.3	5	11	6
	Rakhine	5	47.4	2	1	2
	Karen	3	36.7	2	0	1
	Chin	2	41.5	2	0	0
	Mon	2	31.5	2	0	0
	Others (Kachin, Paoh)	3	61.7	0	2	1
	**Overall**	**37**	**47.1**	**13**	**14**	**10**

GC: Gastric cancer; NUD: Non-ulcerative dyspepsia; PUD: Peptic ulcer disease.

**Table 3:** List of bacterial genera abundances that significantly differed between Orang Asli, Myanmar and modern Malaysian groups diagnosed with NUD.

Genus	Median Relative Abundance (%)			Adjusted *p*-Values		
	OA (*n* = 10)	MYA (*n* = 13)	MM (*n* = 20)	OA vs. MYA	OA vs. MM	MYA vs. MM
*Aquabacterium*	0.69	0	0	0.005	<0.001	N.S
*Asticcacaulis*	0.77	0	0	0.004	<0.001	N.S
*Bosea*	1.24	0	0	0.004	<0.001	N.S
*Brevibacillus*	2.09	0	0	0.004	<0.001	N.S
*Brevundimonas*	1.86	0.01	0	0.004	0.001	N.S
*Caulobacter*	0.72	0	0	0.004	0.001	N.S
*Emticicia*	3.11	0	0	0.004	<0.001	N.S
*Flavobacterium*	7.7	0	0	0.004	<0.001	N.S
*Ideonella*	2.07	0	0	0.004	<0.001	N.S
*Legionella*	0.84	0	0	0.004	<0.001	N.S
*Massilia*	3.1	0	0.05	0.004	<0.001	N.S
*Methyloversatilis*	0.63	0	0	0.004	0.001	N.S
*Phenylobacterium*	2.96	0	0	0.004	<0.001	N.S
*Sphingorhabdus*	2.18	0	0	0.004	<0.001	N.S
*Sulfuritalea*	3.96	0	0	0.004	<0.001	N.S
*Tardiphaga*	0.55	0	0	0.004	<0.001	N.S
*Variovorax*	1.49	0	0	0.004	<0.001	N.S
*Helicobacter*	0.01	0.49	71.69	N.S	0.001	0.01
*Nocardioides*	0.88	0	0.42	0.005	N.S	0.001
*Agrobacterium*	0.62	0	0	0.005	N.S	N.S
*Streptococcus*	0.36	11.06	1.89	0.007	N.S	N.S
*Anaerococcus*	0.01	0.01	0.59	N.S	0.004	N.S
*Corynebacterium*	0.1	0.41	1.6	N.S	0.001	N.S
*Dehalogenimonas*	0.29	0	0	N.S	0.01	N.S
*Desulfatiglans*	1.85	0	0	N.S	<0.001	N.S
*Paludibaculum*	0.71	0	0	N.S	0.006	N.S
*Rothia*	0	0.44	0.33	N.S	0.006	N.S
*Lactobacillus*	0	0.59	0	N.S	N.S	0.004

OA: Orang Asli; MYA: Myanmars; MM: Modern Malaysians; N.S: Not significant.

## Supplementary Materials

The following are available online at https://www.mdpi.com/2076-2607/7/6/174/s1, Table S1, Demographics data and accession numbers; Table S2, OTU table with taxonomic information; Table S3, KEGG pathway abundances predicted based on the gastric microbiome of Orang Asli, Myanmar and modern Malaysian groups; Table S4, Distinguishing KEGG pathways between Orang Asli, Myanmar and modern Malaysians groups; Table S5, Pearson correlation analysis between KEGG pathways and bacterial genera of Orang Asli, Myanmar and modern Malaysian groups.
